# Radiopharmaceuticals for Cancer Imaging and Therapy

**DOI:** 10.3390/pharmaceutics15092262

**Published:** 2023-08-31

**Authors:** Guy Bormans, Frederik Cleeren

**Affiliations:** Laboratory for Radiopharmaceutical Research, Department of Pharmaceutical and Pharmacological Sciences, KU Leuven, 3000 Leuven, Belgium; guy.bormans@kuleuven.be

Nuclear medicine has emerged as a pivotal player in cancer patient care, revolutionizing the way cancer is detected, diagnosed, monitored, and treated. The specific decay characteristics of the radionuclide determine if the radiopharmaceutical can be used for diagnostic (positron emission tomography (PET) and single-photon-emission-computed tomography (SPECT)) or therapeutic (targeted radionuclide therapy (TRT)) purposes. Expression of the molecular target is confirmed during the diagnostic phase, predicting the response to the treatment administered during the therapeutic phase and, afterwards, the effect can be followed-up in downstream diagnostic scans. This concept is called theranostics, see what you treat and treat what you see, and is an excellent example of personalized medicine. As big pharma has stepped into the arena of radiopharmaceuticals for TRT and contributed to the initial success and large investments in this area, a spectacular growth in these radiopharmaceuticals is expected in the near future.

The Special Issue “Radiopharmaceuticals for Cancer Imaging and Therapy” delves into the rapidly evolving landscape of this research field, highlighting the progress made in identifying novel molecular targets, vector molecule design, and radiochemistry using established and upcoming radionuclides and dosimetry to achieve a more specific imaging of cancer targets and improve therapeutic efficacy. In this Special Issue, 137 authors have contributed, with thirteen original research articles and four reviews, providing important insights into the field.

One of the focal points of this Special Issue is the exploration of TRT using high-molar-activity samarium-153, produced by combining the neutron activation of samarium-152 and mass separation of the resulting samarium-153 from carrier samarium-152. Vermeulen et al. from the Belgian Nuclear research center have successfully labeled DOTATATE with ^153^Sm, resulting in [^153^Sm]Sm-DOTATATE, and successfully evaluated the compound to target somatostatin receptors overexpressed in neuroendocrine tumors [1]. This proof-of-concept study shows the potential of mass-separated samarium-153 and could open doors towards the wider application of mass separation in medical isotope production.

Fonge et al., from the university of Saskatchewan, have successfully developed and evaluated [^89^Zr]Zr-matuzumab as a PET probe for the diagnosis/monitoring of response to the treatment of a noncompeting anti-EGFR nimotuzumab antibody drug conjugate (ADC) using mouse colorectal cancer (CRC) xenografts [2]. MicroPET/CT imaging and the biodistribution of [^89^Zr]Zr-matuzumab in mice bearing EGFR-positive xenografts showed a high uptake that could be blocked with the pre-dosing of matuzumab but not with the noncompeting binder nimotuzumab. The simultaneous use of these non-competing antibodies represents a novel approach that holds promise for improving the diagnosis and treatment of EGFR-positive CRC in clinical settings.

Antibodies are interesting vector molecules for the development of novel radiopharmaceuticals, but they have a long blood circulation time and delayed tumor uptake, often limiting their diagnostic applications. Smaller antibody fragments or affibody constructs are excellent platforms for diagnostic radiopharmaceuticals in combination with short-lived PET radionuclides, allowing for good contrast imaging sooner after injection. Dewulf et al., from the university of Antwerp, reported a novel RANKL PET imaging agent, [^64^Cu]Cu-NOTA-denos-Fab, that allows for fast tumor imaging with improved imaging contrast when compared with its antibody counterpart, showing promise as a potential PET RANKL imaging tool for future clinical applications [3]. Researchers from the university of Uppsala further illustrated the potential of radiolabeled affibody molecules for both diagnostic and therapeutic applications, with promising results [4,5,6].

Peptide analogs have also shown great promise as vectors for targeted cancer therapy. The research article by Hörmann et al. investigates the impact of N-terminal peptide modifications on the in vitro and in vivo properties of ^177^Lu-labeled minigastrin analogs targeting CCK2R [7]. This research highlights the importance of molecular design in enhancing the tumor-targeting abilities of peptide analogs, thereby improving the therapeutic potential of radiopeptide-based cancer therapy.

Finally, accurate dosimetry can become a crucial aspect of targeted radionuclide therapy to achieve optimal therapeutic efficacy while minimizing potential side effects. The article “A Review on Tumor Control Probability and Preclinical Dosimetry in TRT” offers a comprehensive review of tumor control probability and preclinical dosimetry techniques, together with their limitations and applications [8]. The conclusion was that additional comprehensive studies at the sub-cellular, cellular, and organ level are essential to experimentally confirm the theoretical basis of TCP models for TRT and to gain further insights into the TRT-specific quantities of these models.

The guest editors of this Special Issue extend their heartfelt gratitude and sincerely appreciate all the authors and reviewers who responded to our invitation. Your willingness to share the outcomes of your outstanding research has greatly enriched this collection, and your contributions have been invaluable in the meticulous evaluation of the manuscripts.
